# TMPRSS12 Functions in Meiosis and Spermiogenesis and Is Required for Male Fertility in Mice

**DOI:** 10.3389/fcell.2022.757042

**Published:** 2022-04-25

**Authors:** Jingjing Zhang, Xinli Zhou, Danyang Wan, Li Yu, Xu Chen, Tong Yan, Zhu Wu, Meimei Zheng, Feng Zhu, Hui Zhu

**Affiliations:** ^1^ State Key Laboratory of Reproductive Medicine, Department of Histology and Embryology, Nanjing Medical University, Nanjing, China; ^2^ Department of Prenatal Diagnosis, Women’s Hospital of Nanjing Medical University, Nanjing Maternity and Child Health Care Hospital, Nanjing, China; ^3^ Reproductive Medicine Center of No. 960 Hospital of PLA, Jinan, China; ^4^ Department of Pathology, The First People’s Hospital of Changzhou, Changzhou, China

**Keywords:** TMPRSS12, meiosis, spermiogenesis, serine protease, male infertility

## Abstract

Serine proteases are involved in many physiological activities as initiators of proteolytic cascades, and some members have been reported to play roles in male reproduction. Transmembrane serine protease 12 (TMPRSS12) has been shown to regulate sperm motility and uterotubal junction migration in mice, but its role in the testis remains unknown. In this study, we verified that TMPRSS12 was expressed in the spermatocytes and spermatids of testis and the acrosome of sperm. Mice deficient in *Tmprss12* exhibited male sterility. In meiosis, TMPRSS12 was demonstrated to regulate synapsis and double-strand break repair; spermatocytes of *Tmprss12* ^−/−^ mice underwent impaired meiosis and subsequent apoptosis, resulting in reduced sperm counts. During spermiogenesis, TMPRSS12 was found to function in the development of mitochondria; abnormal mitochondrial structure in *Tmprss12* ^−/−^ sperm led to reduced availability of ATP, impacting sperm motility. The differential protein expression profiles of testes in *Tmprss12* ^−/−^ and wild-type mice and further molecule identification revealed potential targets of TMPRSS12 related to meiosis and mitochondrial function. Besides, TMPRSS12 was also found to be involved in a series of sperm functions, including capacitation, acrosome reaction and sperm-egg interaction. These data imply that TMPRSS12 plays a role in multiple aspects of male reproduction.

## Introduction

Sperm are the ultimate executors of male reproduction, and a sufficient quantity and quality of sperm guarantee male fertility. Sperm production in the testis, i.e., spermatogenesis, involves spermatogonia self-renewal, spermatocyte meiosis and spermatid differentiation (spermiogenesis), which ultimately form sperm with appropriate morphology, structure, and molecular reserve (Neto et al., 2016). These sperm are then released into the epididymis for further functional maturation. The generation and functional maturation of sperm necessitate a large number of proteins expressed in a certain time sequence and in a specific way to participate or play a regulatory role.

Proteases have attracted the attention of researchers, and they cleave peptide bonds to activate protein precursors and produce various active peptides or functional proteins, which further participate in different physiological processes (Barrett et al., 2001; Barrett, 2004; Chalmel and Rolland, 2015). One of the representatives is serine proteases (PRSSs), which account for approximately one-third of all known proteases (Hedstrom, 2002). Among PRSSs, trypsin-like serine proteases are the largest group, and they are responsible for numerous biological processes, such as digestion, haemostasis, apoptosis, signal transduction, reproduction, and immune response (Hedstrom, 2002; Page and Di Cera, 2008). Many trypsin-like serine proteases have been demonstrated to play important roles in spermatogenesis and/or sperm function. For example, PRSS41, PRSS42 and PRSS43 are involved in spermatocyte meiosis and germ cell apoptosis (Yoneda et al., 2013; Yoneda and Kimura, 2013). PRSS55 is essential for the structural differentiation and energy metabolism of sperm (Shang et al., 2018; Kobayashi et al., 2020; Zhu et al., 2021). PRSS37 affects sperm migration from the uterus into the oviduct and sperm-zona binding (Shen B. et al., 2013).

We focused on a special trypsin-like serine protease, transmembrane serine protease (TMPRSS). Besides a trypsin-like serine protease domain that is essential for catalysis, TMPRSS also contains a transmembrane structure that functions in signal transduction (Hooper et al., 2001). Although the identified TMPRSS members and their reports are far fewer than those of PRSS, the potential role of TMPRSSs in physiology and pathology has been shown (Antalis et al., 2011). Most of the known TMPRSS members are expressed in specific tissues, and their functions have obvious tissue specificity. For example, TMPRSS3 is predominantly expressed in the inner ear. Cell membrane-bound TMRPSS3 is an essential component for hair cell homeostasis and survival, and mutation of the *Tmprss3* gene can cause nonsyndromic recessive deafness (Guipponi et al., 2008; Fasquelle et al., 2011). TMPRSS6 is expressed in the liver and plays an essential role in regulating the expression of the main regulator of iron homeostasis hepcidin; mutations in *Tmprss6* induce high hepcidin levels, which cause iron-refractory iron deficiency anemia (Finberg et al., 2008; Nai et al., 2016; Al-Jamea et al., 2021). TMPRSS10 is most abundant in cardiac myocytes of the atrium and activates atrial natriuretic peptide (ANP) to regulate blood pressure, and deficiency in TMPRSS10 is reported to cause spontaneous hypertension (Yan et al., 2000; Li B. et al., 2017). These results indicate that TMPRSS members specifically expressed in the testis are very likely to be important for spermatogenesis and male reproduction.

To gain insights into the complicated protein networks involved in spermatogenesis and sperm function, human testis and human sperm proteome databases were constructed in our laboratory through the use of a proteomic research platform, and many novel candidate spermatogenesis-/sperm function-related proteins were obtained (Liu et al., 2013; Wang et al., 2013). The TMPRSS family member TMPRSS12 was identified in both the testis and sperm protein profiles, suggesting its potential important role in testicular spermatogenesis and sperm function. According to recently published transcriptomic data, *Tmprss12* was exclusively expressed in testis tissue and located from spermatocytes to round spermatids (da Cruz et al., 2016; Li H. et al., 2017). In addition, we identified 2 heterozygous mutations (c.634G > C [p. Gly212Arg]) in 300 unrelated infertile men with dyszoospermia, and the mutation site was predicted to be potentially deleterious by searching the Polyphen2 and SIFT databases (Supplementary Figure 1). All these results indicated a relationship between TMPRSS12 and male reproduction.

Larasati et al. first explored the function of TMPRSS12 using a *Tmprss12* knockout (KO) mouse model, and they found that *Tmprss12* ^*−/−*^ mice were male sterile and demonstrated that TMPRSS12 regulated sperm motility and ADAM3-related sperm migration to the oviduct (Larasati et al., 2020). The above study focused on the effect of TMPRSS12 on sperm function. Nevertheless, *Tmprss12* expression begins at the stage of zygotene spermatocytes in the testis according to the report of Larasati et al., which is consistent with the reported expressive feature of *Tmprss12* obtained by transcriptome sequencing (da Cruz et al., 2016; Li B. et al., 2017). Thus, TMPRSS12 might function in spermatogenesis. Therefore, our study aimed to use *Tmprss12* ^*−/−*^ mice to reveal the function and mechanism of TMPRSS12 in spermatogenesis. Our results provide detailed information that TMPRSS12 affects spermatogenesis, which elucidate the role of TMPRSS12 in male reproduction more comprehensively.

## Materials and Methods

### Animals

*Tmprss12* ^*−/−*^ mice were generated on the C57BL/6 background via Cas9/RNA-mediated gene targeting as described previously (Shen C. et al., 2013). All the mice were housed under specific pathogen free condition with unlimited access to food and water. The constant room was maintained at the temperature of 22–24°C with a light:dark cycle of 12:12. All the research involving animal experiments were approved by the ethics committees of Nanjing Medical University.

### RNA Isolation, cDNA Synthesized, PCR and Quantitative Real-Time PCR Analyses

Total RNA was extracted from the sample by using Trizol reagent (Invitrogen, 15596-026) according to the manufacturer’s instructions. mRNA was reverse transcribed to cDNA using HiScript II Q Select RT SuperMix for qPCR (Vazyme, R232-01). PCR (polymerase chain reaction) was performed using 2 × Taq Plus Master Mix (Vazyme, P212-01), with primers specific for mouse *Tmprss12*. The PCR products were analyzed by agarose gel electrophoresis using mouse *Actin* as an internal control. Quantitative real-time PCR of cDNA was carried out using AceQ qPCR SYBR Green Master Mix (Vazyme, Q141-02) according to the manufacturer’s instructions with an ABI Q5 real-time PCR System (Applied Biosystems, Thermo Fisher Scientific). The primer sequences used for these experiments are listed in Supplementary Table 1.

### Western Blot Analysis

Protein extracts from mouse tissues or sperms were separated on 10% SDS-PAGE gels and transferred onto nitrocellulose membranes (Bio-Rad, 1620177). Membranes were blocked with 5% nonfat milk and then incubated overnight with the primary antibodies: anti-TMPRSS12 (Santa Cruz Biotechnology, sc-249059; 1:500), anti-TEKTIN-T (Proteintech, 13518-1-AP; 1:500), anti-IQCG (Bioss, bs-9022R; 1:500), anti-CLIP170 (Proteintech, 23839-1-AP; 1:2,000), anti-MEIG1 (Bioss, bs-18778R; 1:500), anti-AGFG1 (ZENBIO, 513500; 1:1,000), anti-KLC3 (Santa Cruz Biotechnology, sc-398332E-7; 1:500), anti-COX2 (ZENBIO, 381136; 1:1,000), anti-COX3 (ZENBIO, 381270; 1:1,000), anti-MTATP (Proteintech, 55313-AP; 1:500), anti-MTCYB (Proteintech, 55090-1-AP; 1:500), anti-BAX (Proteintech, 50599-2-Ig; 1:5,000), anti-BCL2 (Proteintech, 12789-1-AP; 1: 2,000), anti-CASPASE3 (Proteintech, 19677-1-AP; 1:1,000), anti-HELLS (Abclonal, A5831; 1:1,000), anti-RAD51 (Abclonal, A6268; 1:500), anti-BCCIP (Abclonal, A8586; 1:500), anti-MGST1 (Abclonal, A16399; 1:1,000), anti-PGAM2 (Abclonal, A7917; 1:1,000), anti-β-ACTIN (Biogot, AP0060; 1:10,000), anti-α-TUBULIN (Biogot, BS1482M; 1:10,000). The washed membranes were incubated with the secondary antibodies according to the appropriate concentration. The specific protein bands were detected by an ECL kit (Thermo, 32109) and Bio-Rad gel imaging system.

### Assessment of Fertility

Three adult *Tmprss12* ^−/−^ mice and wild-type (WT) mice were used for fertility tests. Each male was matched with two females. Male and female mice were paired in this manner for 6 months and the number of live-born pups for each pairing was recorded.

### Computer Assisted Sperm Analysis

The sperm count and motility was quantified by Computer Assisted Sperm Analysis detection with the IVOS II™ system (Hamilton Thorne, United States). Briefly, sperm from *Tmprss12* ^−/−^ and WT mice were extracted from the cauda epididymis and incubated in human tubal fluid (HTF) culture medium (Easy Check, M1130) at 37°C. The sperm suspension was added to a counting chamber for analysis.

### Histological Analysis

Testes from adult mice were fixed in modified Davidson’s fluid, embedded in paraffin and cut into 5-µm sections. Periodic acid-Schiff (PAS) staining was performed for testis sections according to the manufacturer’s protocol (Thermo, 87007) to determine the stage of spermatogenesis. Each stage with a distinct ordering of cell associations along the length of the seminiferous tubules was designated by Roman numerals, and approximately 200 tubules from each section were analyzed under microscopy (ZEISS, Germany). For sperm morphology analysis, sperm was washed three times in phosphate buffered saline (PBS) and transferred to slides. After immobilization with 4% PFA, sperm was stained with hematoxylin-eosin (HE) (Beyotime, C0105S). At least 200 sperm cells were analyzed per sample.

### Immunofluorescence Analysis

The prepared sections were blocked in 1% bovine serum album for 2 hours and then incubated with the goat polyclonal anti-TMPRSS12 (1:100, sc-249059; Santa Cruz Biotechnology) overnight at 4°C. The sections were incubated with fluorescently labeled secondary antibodies at room temperature for 2 h. After being counterstained with DAPI, the slides were viewed with a LSM700 confocal microscope (Zeiss, Germany).

### Terminal Deoxynucleotidyl Transferase dUTP Nick End Labeling Assays

Apoptosis detection of spermatogenic tubules and cells was conducted using a terminal deoxynucleotidyl transferase nick-end labeling (TUNEL) BrightRed Apoptosis Detection Kit (Vazyme, A113-01), following the manufacturer’s directions. Briefly, the testis sections were equilibrated with TdT buffer for 20 min at room temperature. TdT buffer was removed and terminal transferase reaction mix was added. The reaction was performed for 1 h at 37°C. Sections were washed with PBS and then counterstained with DAPI. Slides were viewed under a LSM700 confocal microscope (Zeiss, Germany).

### MI Chromosome Spread

Chromosome spreads of prophase I spermatocytes were performed as previously described (Peters et al., 1997; Kolas et al., 2005). In brief, seminiferous tubules were separated, cut into pieces with scissors, and incubated in hypotonic buffer (0.45% NaCl) for 40 min. Cell suspensions were fixed with 1% paraformaldehyde containing 0.15% Triton X-100 and 0.5 M sodium borate and then air-dried on slides. Samples were blocked with 1x ADB (1% normal donkey serum, 0.03% BSA, and 0.05% Triton X-100) for 1 h and then incubated with primary antibodies at 37°C for 12–16 h. After being blocked with 1x ADB for 5–6 h at room temperature, the samples incubated in fluorescently labeled secondary antibodies at 37 °C for 1.5 h. The slides were viewed under a LSM700 confocal microscope (Zeiss, Germany). Primary antibodies were as follows: SYCP1 (1:100, NB300-229; Novus Biologicals), SYCP3 (1:500, ab97672; Abcam), γH2AX (1:500, ab2893; Abcam).

### Electron Microscopy Observation

Sperm samples separated from cauda epididymis were fixed in 0.1 M phosphate buffer (pH7.3) containing 2.5% glutaraldehyde and 4% (vol/vol) glutaraldehyde with 2% (wt/vol) OsO4 and then embedded in Araldite. Transmission electron microscopy was performed by the Electron Microscopy Laboratory of Nanjing Medical University. Transmission electron microscopy imaging was conducted on a JEM-1010 transmission electron microscope (JEOL, United States, Inc.).

### Sperm ATP Assay

According to the manufacturer’s instructions (Beyotime, S0027), sperm samples were washed twice, resuspended in lysis buffer, vortexed and then placed on ice. ATP was measured by luminometric methods using commercially available luciferin/luciferase reagents in luminometer (TD-20/20, Turner Designs). An average of 5 × 10^7^ sperm were used for ATP analysis.

### Mitochondrial Membrane Potential Assay

The JC-1 mitochondrial membrane potential detection kit (Beyotime, C2003S) was used to determinate the MMP. Briefly, sperm were incubated with JC-1 staining working fluid in the dark at 37°C for 20 min. After washed by PBS, the sperms were analyzed by an LSM700 confocal microscope (Zeiss, Germany) and flow cytometer. JC-1 monomers give green fluorescence while JC-1 aggregates produce red fluorescence. MMP was analyzed by relative ratio of red/green fluorescence.

### Assays of Extracellular Acidification and Oxygen Consumption

Extracellular acidification and oxygen consumption were analysed by a glycolysis assay kit (Abcam, ab197244) and extracellular oxygen consumption assay kit (Abcam, ab197243), respectively, according to the manufacturer’s instructions. Briefly, sperm were plated at 50,000 cells per well in a 96-well plate, and then test reagent was added to the sample wells. For the oxygen consumption assay, two drops of prewarmed high-sensitivity mineral oil were added to each well before measurement. Fluorescence (Ex 360 nm Em 620 nm for the extracellular acidification assay; Ex 380 nm Em 645 nm for the oxygen consumption assay) was measured every 6 min for 1 h by using a microplate reader (BioTek Synergy Mx, United States).

### 2-D Gel Electrophoresis Combined with Matrix-Assisted Laser Desorption/Ionization Time-of-Flight Tandem Mass Spectrometry (MALDI-TOF/TOF MS/MS)

Testes proteins were extracted from WT and *Tmprss12* ^*−/−*^ mice in lysis buffer. The protein was loaded and separated by gel rehydration on 24-cm immobilized, PH3-10, nonlinear gradient strips for 2-D according to the previously described (Guo et al., 2010). After isoelectric focusing carried out in an IPGphor apparatus (GE Healthcare, San Francisco, CA, United States), the samples for second-dimension were separated in 12% polyacrylamide gels by an Ettan-Dalt 6 system (GE Healthcare). Subsequently, the gels were stained with silver staining. The sliver-stained gels were scanned and analyzed using 2D Elite Image Master software (GE Healthcare). The protein spots, which were shown >2-fold difference (*p* < 0.05, Student’s *t*-test) between WT and *Tmprss12* ^−/−^ testes, were excised, dehydrated in ACN, and dried at room temperature. The protein spots were digested according to our previous described (Guo et al., 2010). The digests were immediately spotted onto 400-µm anchor chips (Bruker Daltonics). The protein spots were subsequently subjected to identification by MALDI-TOF/TOF analysis. All the samples were analyzed in a time-of-flight Biflex IV mass spectrometer (Bruker Daltonics). Tandem mass spectra were given by an Ultraflex II mass spectrometer (Bruker Daltonics) in LIFT mode and processed using FlexAnalysis software (version 2.4; Bruker Daltonics). Each acquired tandem mass spectrometry (MS/MS) queries were performed using the Mascot (version 2.1, Matrix Science, London, United Kingdom) search engine in the Uniport-House mouse database. All MS/MS identifications by Mascot were manually verified for spectral quality and y and b ion series matches.

### Statistical Analysis

All experiments were repeated at least three times. Significance was tested by using Two-way ANOVA or Student’s *t* test using Prism software (GraphPad Software, La Jolla, CA, United States). Values of *p* < 0.05 were considered to be statistically significant. All data are reported as means ± the standard deviation of the mean (SD).

## Results

### TMPRSS12 Is Specifically Expressed in Testicular Spermatocytes and Spermatids as Well as Sperm

**FIGURE 1:**
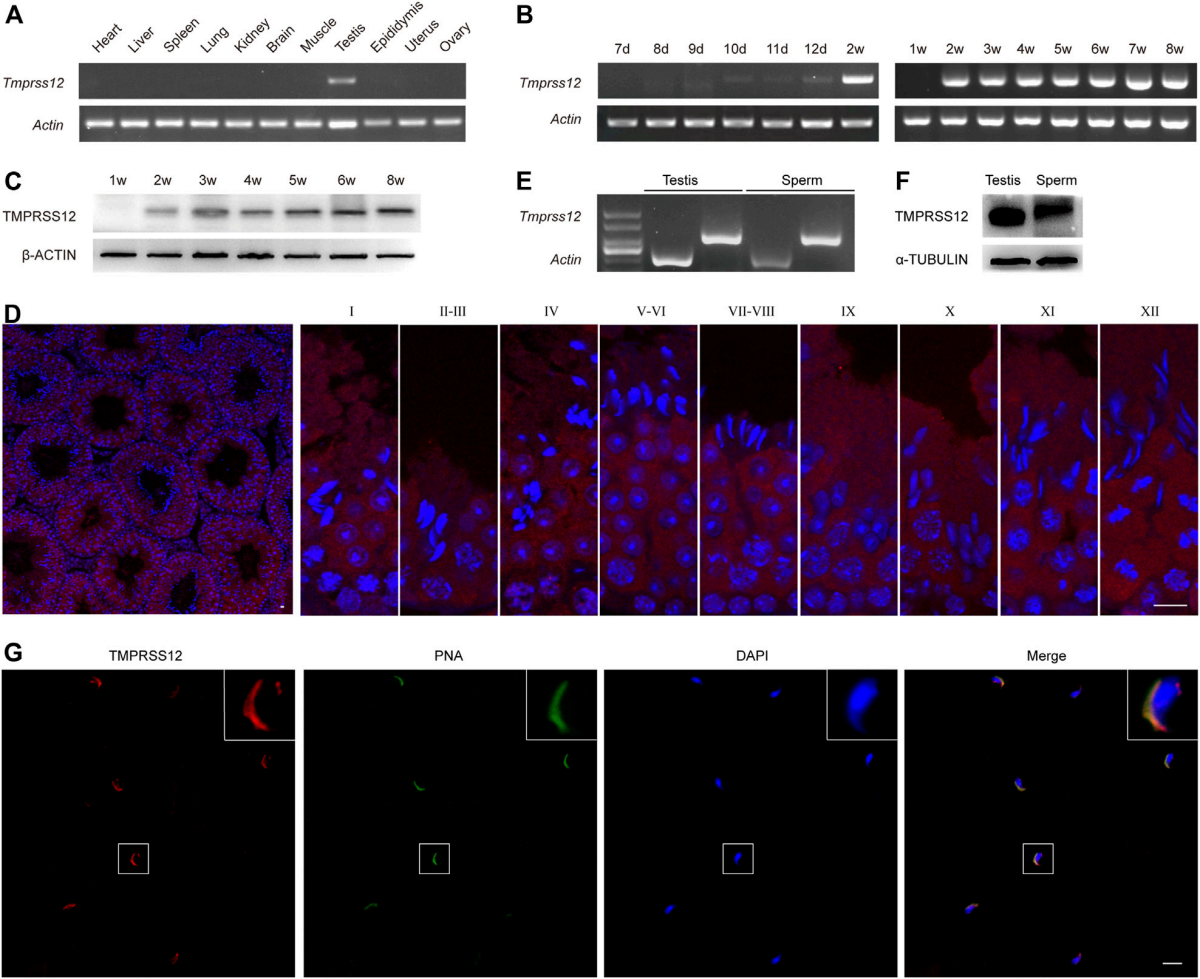
TMPRSS12 is expressed in testicular spermatocytes and spermatids as well as sperm. **(A)** Expression of *Tmprss12* mRNA in various tissues from adult mice was detected by RT-PCR. TMPRSS12 was specifically expressed in the testis. *Actin* was used as an internal control. **(B)** Expression of testicular *Tmprss12* mRNA at the indicated time points after birth was examined by RT-PCR, showing that its expression first appeared in 10-day-old mice. *Actin* was used as an internal control. **(C)** Western blot of TMPRSS12 from the testes of mice at different weeks of age. TMPRSS12 was observed in 2-week-old mice and continued until adulthood. β-ACTIN was used as an internal control. **(D)** Immunofluorescence staining of testis sections from adult mice for TMPRSS12 (red), with nuclei counterstained by DAPI (blue). Each image exhibits a stage of the seminiferous epithelial cycle, and they are denoted by Roman numerals at the top of each image. The expression of TMPRSS12 initiated from spermatocytes and remained until step 12 elongated spermatids. Scale bar: 10 µm. **(E)** Expression of *Tmprss12* was examined by RT-PCR using RNA isolated from mouse sperm. *Actin* was used as an internal control. **(F)** Western blot of TMPRSS12 in mouse sperm. α-TUBULIN was used as an internal control. **(G)** Immunofluorescence staining of mouse sperm for TMPRSS12 (red). The nuclei and acrosomes of sperm were labelled with DAPI (blue) and PNA (green), respectively. The images in the upper right corner are enlarged views of the sperm. Scale bar: 20 µm.

The expression of *Tmprss12* was assayed by RT-PCR performed with 11 mouse tissues, including heart, liver, spleen, lung, kidney, brain, muscle, testis, epididymis, uterus and ovary tissues. As shown in Figure 1A, *Tmprss12* was exclusively expressed in testis tissue. By PCR and western blot detection, we found at the mRNA and protein levels that TMPRSS12 was first detected in the testes of 10-day-old mice, and its expression remained constant after 14 days of age (Figures 1B,C). We further detected the expression characteristics of TMPRSS12 in mouse testes by immunofluorescence analysis. The results demonstrated TMPRSS12 expression in germ cells within the seminiferous tubules and showed that its expression was initiated from spermatocytes and remained until step 12 elongated spermatids (Figure 1D). We also confirmed the expression of TMPRSS12 in mature sperm by PCR and western blot analysis (Figures 1E,F), and the immunofluorescence results showed positive signals of TMPRSS12 located in the acrosome (Figure 1G). The above results suggested the potential important role of TMPRSS12 in male reproductive function, which very likely influenced meiosis, spermiogenesis and acrosome-related sperm function.

### Targeted Disruption of TMPRSS12 Results in Male Infertility

**FIGURE 2:**
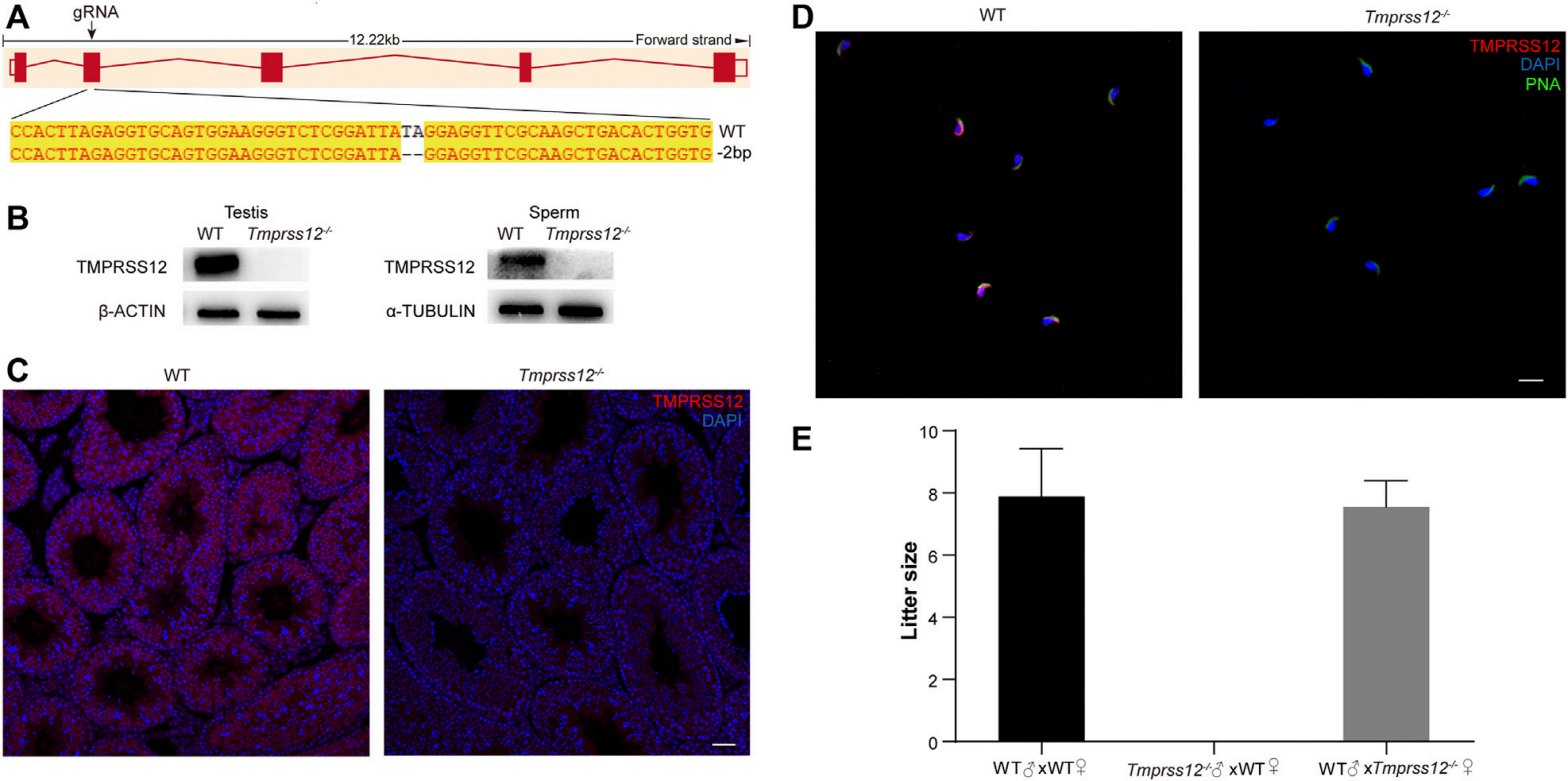
*Tmprss12* knockout mice display male sterility. **(A)** Schematic strategies for the generation of *Tmprss12* ^−/−^ mice using CRISPR/Cas9 technology. Image of Sanger sequencing results showing a 2-bp deletion in exon 2. **(B)** Western blot of the testes and sperm from WT and *Tmprss12* ^−/−^ mice to verify the validation of *Tmprss12* knockout. β-ACTIN and α-TUBULIN were used as internal controls. **(C)** TMPRSS12 expression in the testes of WT and *Tmprss12* ^−/−^ mice was evaluated through immunofluorescence analysis. No TMPRSS12 signal was detected in *Tmprss12* ^−/−^ mice. Scale bar: 50 µm. **(D)** TMPRSS12 expression in the sperm of WT and *Tmprss12* ^−/−^ mice was determined by immunofluorescence analysis. A positive signal was not observed in *Tmprss12* ^−/−^ sperm. Scale bar: 20 µm. **(E)** Number of pups per litter produced by *Tmprss12* ^−/−^ mice and WT mice. *Tmprss12* ^−/−^ male mice are infertile (*n* = 3). Data are the mean ± s.d.

To investigate whether TMPRSS12 is required for male fertility, *Tmprss12* knockout mice were generated using the CRISPR/Cas9 system. The injection of a single guide RNA targeting *Tmprss12* exon 2 produced knockout mice harbouring a 2-bp deletion. A representative image of the Sanger sequencing results for the verification of the *Tmprss12* ^***−/−***^ mouse is shown in Figure 2A. We confirmed the lack of TMPRSS12 in the testes and sperm of *Tmprss12* ^***−/−***^ mice by western blot and immunofluorescence assays (Figures 2B–D).

Mating tests were performed and continuous monitoring was conducted for 6 months to analyse the fertility of the mice. The female *Tmprss12* ^***−/−***^ mice were fertile with litter sizes comparable to those of WT females. However, *Tmprss12* ^***−/−***^ males were infertile and produced no litters (Figure 2E). These results suggest that TMPRSS12 is required for male reproduction.

### *Tmprss12* ^−/−^ Mice Have Decreased Sperm Count and Reduced Sperm Motility

**FIGURE 3:**
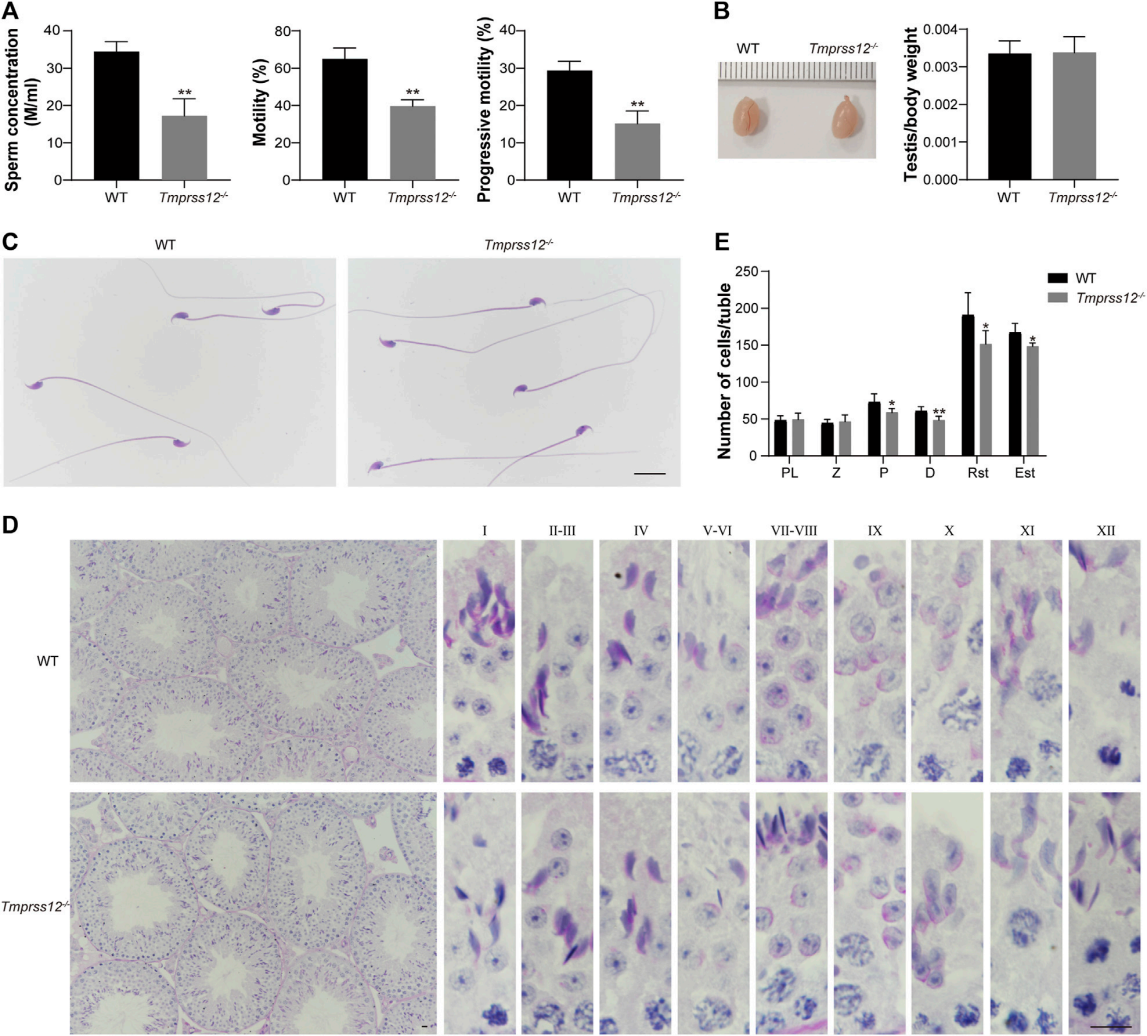
*Tmprss12* knockout mice exhibit oligoasthenospermia, with decreased spermatogenic cells in testes. **(A)** Computer assisted sperm analysis (CASA) showed that the sperm concentration was reduced and motility and progressive motility were decreased in *Tmprss12* ^−/−^ mice (*n* = 3). Data are the mean ± s.d. ^∗∗^ *p* < 0.01. **(B)** Gross morphology of testes from WT and *Tmprss12* ^−/−^ mice and relative testis weight to body weight in the two groups showed no significant difference (*n* = 3). Data are the mean ± s.d. **(C)** The sperm morphology of WT and *Tmprss12* ^−/−^ mice. The sperm of *Tmprss12* ^−/−^ mice showed no obvious abnormality. Scale bar: 20 µm. **(D)** Periodic acid-Schiff staining of testis sections from WT and *Tmprss12* ^−/−^ mice. Complete spermatogenic tubules and orderly arranged spermatogenic cells could be seen in the testes of *Tmprss12* ^−/−^ mice. Scale bar: 10 µm. **(E)** Reduced number of spermatogenic cells in *Tmprss12* ^−/−^ testes. Numbers of spermatogenic cells per tubule are shown. 10 tubules cross sections for each stage were counted. Compared with the WT, the number of pachytene spermatocytes, diplotene spermatocytes and spermatids of *Tmprss12* ^−/−^ mice decreased significantly (*n* = 6). Data are the mean ± s.d. *p < 0.05, **p < 0.01.

To explore the possible reasons for infertility in *Tmprss12* ^*−/−*^ mice, we first detected the sperm parameter by Computer Assisted Sperm Analysis (CASA). The results showed that the sperm count and motility were significantly decreased in *Tmprss12* ^*−/−*^ mice compared to WT mice (Figure 3A).

The weight and size of the testis did not differ significantly, and the gross morphology and histology of the testis and sperm were similar between the WT and *Tmprss12* ^*−/−*^ mice (Figures 3B–D). Moreover, we paid close attention to the spermatogenesis of *Tmprss12* ^*−/−*^ testes by counting spermatogenic cells at all stages (Figure 3E). The counts of pachytene spermatocytes, diplotene spermatocytes, round spermatids and elongated spermatids all decreased significantly in *Tmprss12* ^*−/−*^ mice compared with WT mice, which probably led to the decreased sperm count in *Tmprss12* ^*−/−*^ mice.

### Increased Apoptosis and Disrupted Meiosis of Spermatocytes in the Testes of *Tmprss12* ^−/−^ Mice

**FIGURE 4:**
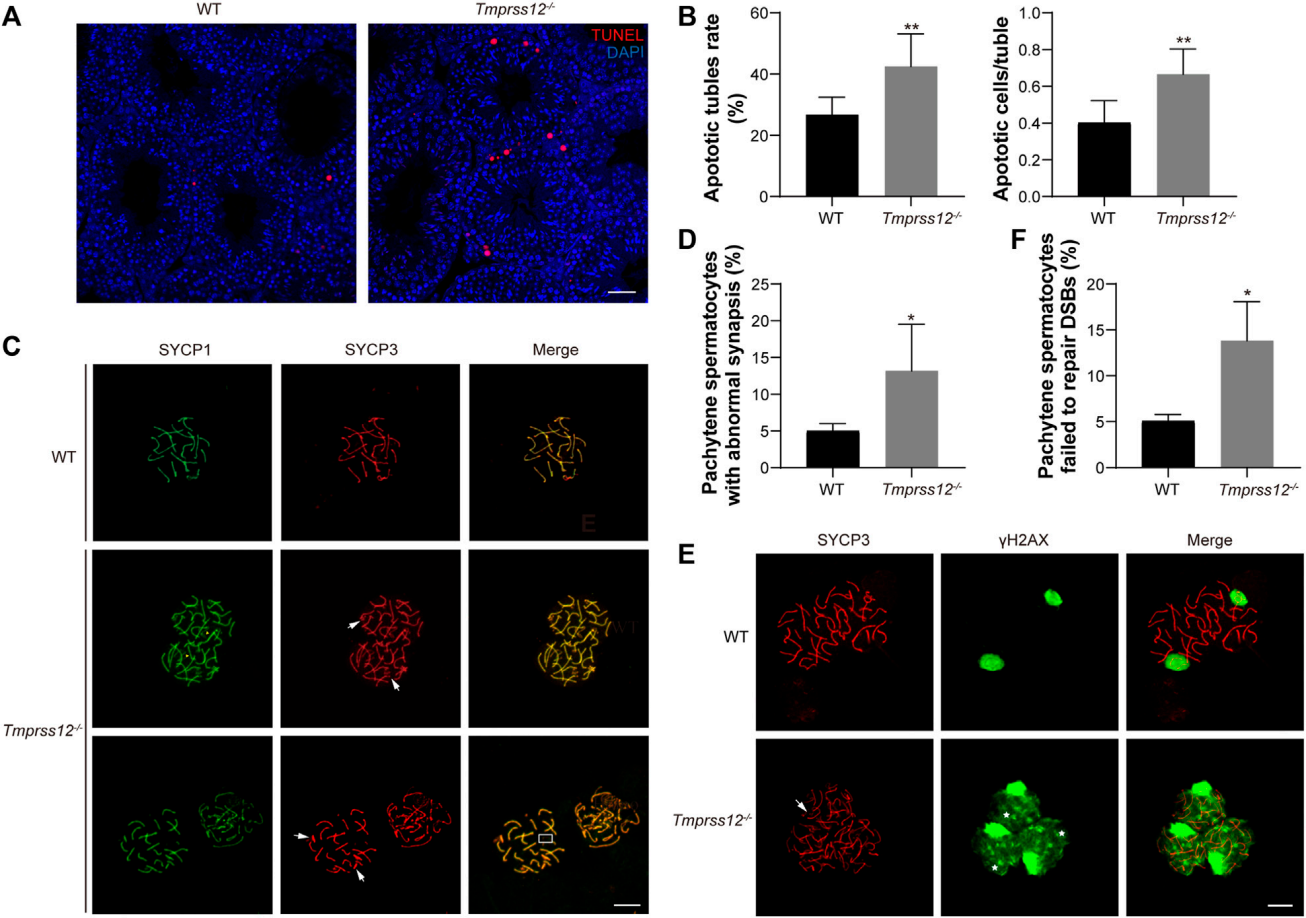
*Tmprss12* deletion leads to damaged synapsis and DSB repair in meiosis. **(A)** Terminal deoxynucleotidyl transferase nick-end-labelling (TUNEL) staining (red) in the testes. Apoptosis in *Tmprss12* ^−/−^ mice was significantly increased, and the apoptotic cells were mainly spermatocytes. Nuclei are stained with DAPI (blue). Scale bar: 50 µm. **(B)** Analysis of the apoptotic tubule rate and the number of apoptotic cells per tubule. All spermatogenic tubules from testis sections of the same size in two groups were observed. The data showed increased apoptosis in *Tmprss12* ^−/−^ mouse testes (*n* = 3). Data are the mean ± s.d. ^∗∗^ *p* < 0.01. **(C)** Immunofluorescence staining of SYCP1 and SYCP3 on spermatocyte spreads produced from WT and *Tmprss12* ^−/−^ mouse testes. Chromothripsis (white arrows), no synapsed sex chromosomes (boxed area), and abnormal SYCP1 signals in non-PAR (yellow arrows) were observed in *Tmprss12* ^−/−^ mice. Scale bars: 10 µm. **(D)** Percentage of pachynema spermatocytes with abnormal synapses. The quantitative value was increased in *Tmprss12* ^−/−^ mice (*n* = 3). Data are the mean ± s.d. ^∗^ *p* < 0.05. **(E)** Immunofluorescence staining of SYCP3 and γH2AX on spermatocyte spreads produced from adult WT and *Tmprss12* ^−/−^ mouse testes. In *Tmprss12* ^−/−^ images, white arrows indicate chromothripsis, and asterisks indicate the abnormal γH2AX signal. Scale bars: 10 µm. **(F)** Statistical analysis showing that the percentage of pachytene spermatocytes with abnormal DSB repair was increased compared with that in WT mice (*n* = 3). Data are the mean ± s.d. ^∗^ *p* < 0.05.

To assess the reasons for the decrease in germ cells, we first evaluated the state of spermatogenic cells in the testes of *Tmprss12* ^−/−^ and WT mice. Terminal deoxynucleotidyl transferase nick-end labelling (TUNEL) assays were performed to detect apoptotic cells in the testes of these two groups. We found that the numbers of apoptotic germ cells increased remarkably in *Tmprss12* ^*−/−*^ mice compared with WT mice and the apoptotic cells were mainly spermatocytes (Figures 4A,B).

In spermatogenesis, meiosis is an important biological process that produces haploid germ cells. Abnormal meiosis interferes with the spermatocyte state and affects the progress of spermatogenesis. Based on our findings that spermatocyte apoptosis increased and the number of spermatogenic cells after the zygotene stage decreased, it is reasonable to speculate that TMPRSS12 may be involved in meiosis. Therefore, we examined the key biological events of meiosis in *Tmprss12* ^*−/−*^ testes. We assessed chromosomal synapsis by immunofluorescence analysis using antibodies against SYCP1 and SYCP3, which are components of the synaptonemal complex (SC). Chromosome spreads from WT testes showed that the fluorescence signal of anti-SYCP3 in the pachytene stage completely overlapped with that of anti-SYCP1 around the SC, including 19 pairs of autosomes and partial synapsed sex chromosomes in the pseudoautosomal region (PAR). In the pachytene spermatocytes of *Tmprss12* ^*−/−*^ testes, chromothripsis, no synapsed sex chromosomes, and abnormal SYCP1 signals in non-PAR were observed (Figure 4C). We quantified the spermatocytes of WT and *Tmprss12* ^*−/−*^ testes and found that spermatocytes with abnormal synapses were significantly higher in *Tmprss12* ^*−/−*^ mice than in WT mice (Figure 4D).

In addition, we monitored the meiotic recombination of homologues in spermatocytes. Recombination is initiated by DNA double-strand break (DSB) formation in leptotene spermatocytes. DSBs could be marked by the presence of phosphorylated H2AX (γH2AX). In normal meiosis, γH2AX is present on autosomal chromatin and distributed throughout the nucleus in leptotene spermatocytes, persists into the zygotene stage and is restricted to only the sex chromosomes at the pachytene stage. As shown in Figure 4E, γH2AX disappeared from autosomes following meiotic DSB repair and was restricted to the sex chromosomes in the pachytene stages of WT spermatocytes, but the γH2AX signal was distributed in *Tmprss12* ^*−/−*^ pachytene spermatocytes and was not restricted to sex chromosomes, suggesting failure in the repair of meiotic DSBs. Moreover, the results of quantifying the abnormal spermatocytes in the two groups were significantly different (Figure 4F).

### Increased Sperm Apoptosis in *Tmprss12* ^−/−^ Mice

**FIGURE 5:**
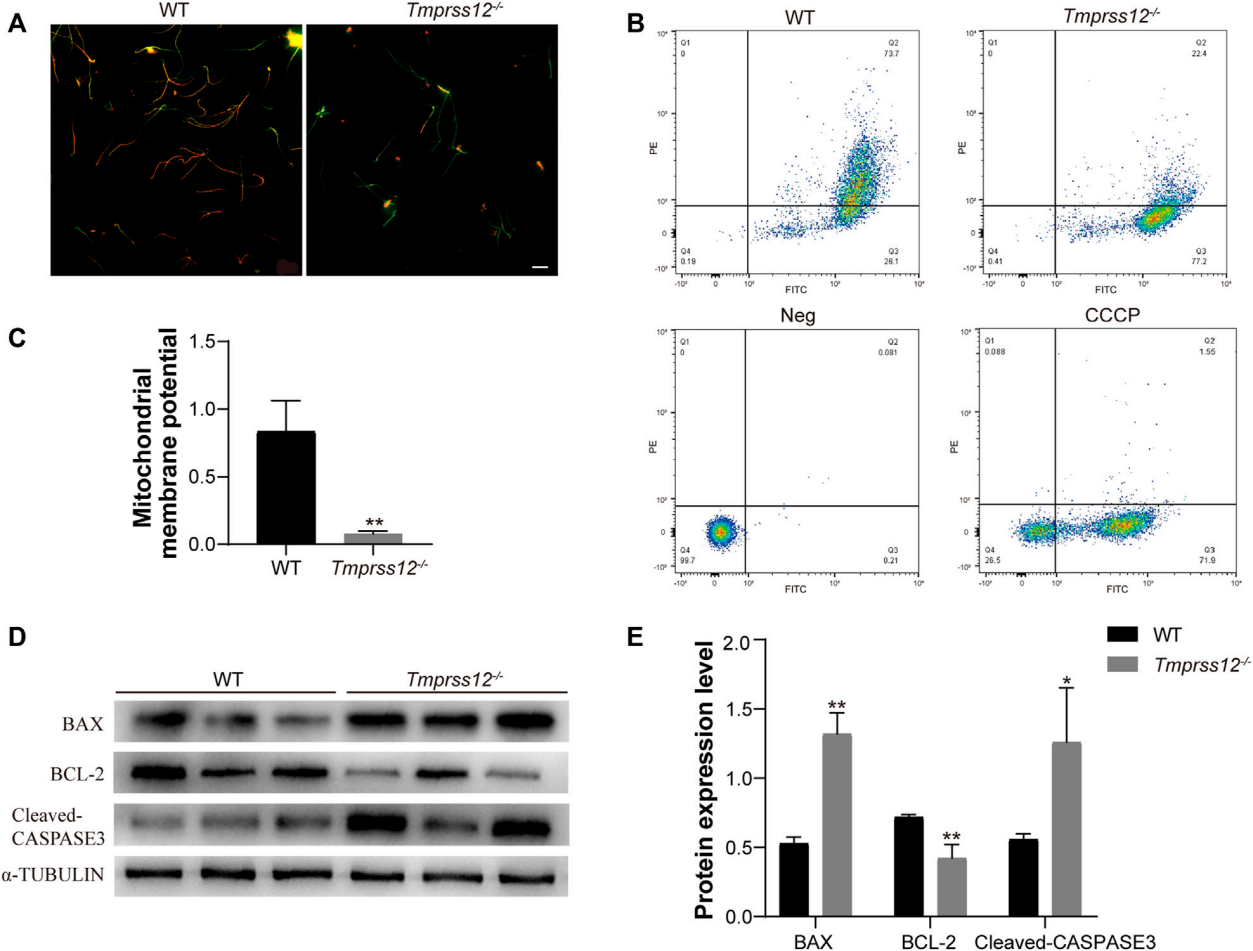
*Tmprss12* deletion causes increased sperm apoptosis. **(A)** Fluorescence microscopy observation of sperm after JC-1 staining between WT and *Tmprss12* ^−/−^ mice. The red fluorescent sperm were normal, and the green sperm had a low mitochondrial membrane potential (MMP). Scale bars: 20 µm. **(B)** Sperm from WT and *Tmprss12* ^−/−^ mice stained with JC-1 dye were checked for their corresponding MMP through flow cytometry analysis. Q2, red-stain sperm; Q3, green-stain sperm. Neg and carbonyl cyanide 3-chlorophenylhydrazone (CCCP) were used as a negative control and positive control, respectively. **(C)** MMP was analysed by the relative ratio of red/green fluorescence. The sperm of *Tmprss12* ^−/−^ mice showed low MMP levels (*n* = 3). Data are the mean ± s.d. ^∗^ *p* < 0.05. **(D)** Western blot of apoptosis-related molecules from sperm of WT and *Tmprss12* ^−/−^ mice. α-TUBULIN was used as an internal control. **(E)** Grey intensity analysis showing the expression level of the above markers. The expression levels of BAX and CASPASE3 were increased while BCL-2 was decreased in *Tmprss12* ^−/−^ mice (*n* = 3). Data are the mean ± s.d. ^∗^ *p* < 0.05.

We further explored whether the apoptotic state of sperm was also involved in the decreased sperm count of *Tmprss12* ^−/−^ mice. Mitochondrial membrane potential (MMP) reduction is an important indicator of early apoptosis. Therefore, we examined the MMP of sperm in *Tmprss12* ^−/−^ mice. JC-1 dye was used to monitor the MMP, which was lower in *Tmprss12* ^−/−^ mice than WT mice (Figures 5A–C). Furthermore, we detected key molecules in classic apoptosis pathways, including BAX, BCL-2 and CASPASE3 (Yang and Cortopassi, 1998; Antonsson et al., 2000; Barroso et al., 2006; Brunelle and Letai, 2009). The expression levels of BAX and CASPASE3 were increased while that of BCL-2 was decreased in *Tmprss12* ^−/−^ mice, which indicated increased sperm apoptosis when TMPRSS12 was deficient (Figures 5D,E).

### Abnormal Mitochondrial Structure and Impaired ATP Synthesis in the Sperm of *Tmprss12* ^−/−^ Mice

**FIGURE 6:**
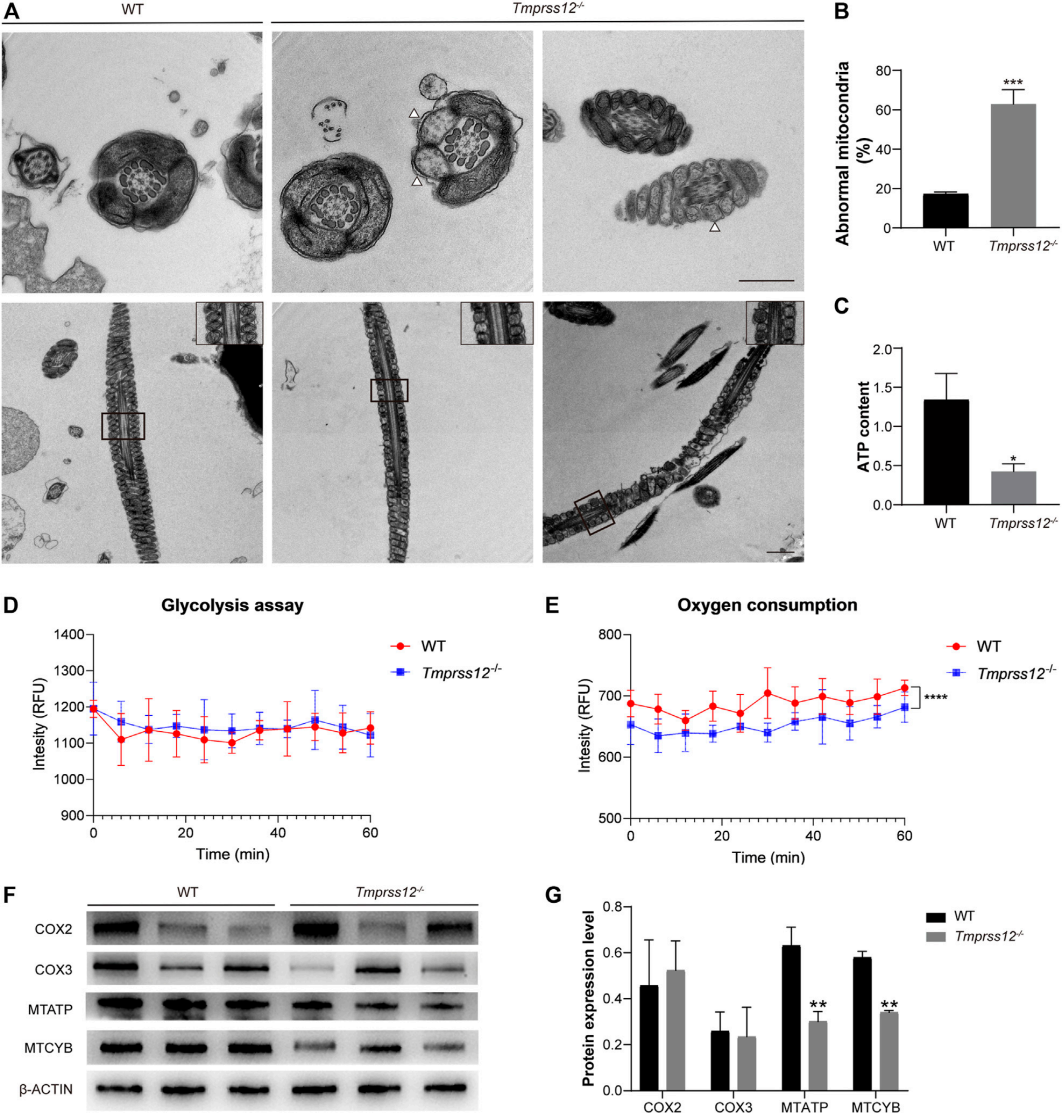
Impaired mitochondrial structure and function in the sperm of *Tmprss12* ^−/−^ mice. **(A)** Transmission electron microscopy (TEM) images of the sperm ultrastructure are shown, revealing blurred or absent inner mitochondrial membrane (IMM) cristae (white arrows). The images in the upper right corner of the second row are enlarged views of the mitochondria. Scale bars: 1 µm. **(B)** Percentage of mitochondria with an abnormal structure. The percentage was increased in *Tmprss12* ^−/−^ mice (*n* = 4). Data are the mean ± s.d. ^∗∗∗^ *p* < 0.001. **(C)** Tested ATP contents of sperm between WT and *Tmprss12* ^−/−^ mice. There were no energy substrates in the sperm isolation buffer and background ATP was consumed before detection. The ATP level was significantly lower in *Tmprss12* ^−/−^ mice (*n* = 3). Data are the mean ± s.d. ^∗^ *p* < 0.05. **(D)** Glycolysis assay measured as cytoplasmic acidification. Sperm of WT and *Tmprss12* ^−/−^ mice were extracted from the cauda epididymis and suspended in respiration buffer, followed by glycolysis measurement for 60 min via fluorescent emission. There was no significant difference in glycolysis between sperm of WT and *Tmprss12* ^−/−^ mice (*n* = 3). Data are the mean ± s.d. **(E)** Oxygen consumption analysis. Sperm of WT and *Tmprss12* ^−/−^ mice were extracted from the cauda epididymis and suspended in PBS. Then fluorescent emission was measured and compared during 60 min via fluorescence spectroscopy. *Tmprss12* ^−/−^ sperm showed lower oxygen consumption than WT sperm (*n* = 3). Data are the mean ± s.d. ****p < 0.0001. **(F)** Western blot of key markers in the mitochondrial electron transfer chain from sperm of WT and *Tmprss12* ^−/−^ mice. β-ACTIN was used as an internal control. **(G)** Grey intensity analysis showing the expression level of the above markers. The expression levels of MTATP and MTCYB were decreased in *Tmprss12* ^−/−^ mice (*n* = 3). Data are the mean ± s.d. **p < 0.01.

The CASA analysis revealed that the sperm motility of *Tmprss12* ^−/−^ male mice was significantly lower than that of WT mice. To explore the reason for the decreased sperm motility, we further observed the ultrastructure of sperm in *Tmprss12* ^*−/−*^ and WT mice under a transmission electron microscope. The results showed that the inner mitochondrial membrane cristae were blurred or absent in *Tmprss12* ^−/−^ mice. According to the statistical analysis, the number of sperms with abnormal mitochondria in *Tmprss12* ^*−/−*^ mice was significantly higher than that in WT mice (Figures 6A,B).

Mitochondria are the energy metabolism centres of cells and the main sites of oxidative phosphorylation for producing ATP. Thus, we detected the sperm ATP content, glycolysis, oxygen consumption and the key markers of the oxidative phosphorylation process in mitochondria. The decreased ATP content in the sperm of *Tmprss12* ^*−/−*^ mice is shown in Figure 6C. Extracellular acidification revealed that the sperm of *Tmprss12* ^−/−^ mice had glycolysis levels similar to those of WT mice (Figure 6D). However, the oxygen consumption assay showed that the oxygen consumption of sperm of *Tmprss12* ^−/−^ mice was reduced compared with that of the sperm of WT mice (Figure 6E). COX2, COX3, MTATP and MTCYB are the key markers of the oxidative phosphorylation process in mitochondria (Feng et al., 2008; Heidari et al., 2016). The mRNA and protein levels of MTATP and MTCYB in the sperm of *Tmprss12* ^−/−^ mice were reduced compared with those in WT mice (Figure 6F,G; Supplementary Figure 2). The above results suggested that an abnormal ultrastructure of mitochondria and impaired ATP synthesis in sperm are potential causes of decreased sperm motility.

### Impaired Mitochondrial Development During Spermiogenesis in *Tmprss12* ^−/−^ Mice

**FIGURE 7:**
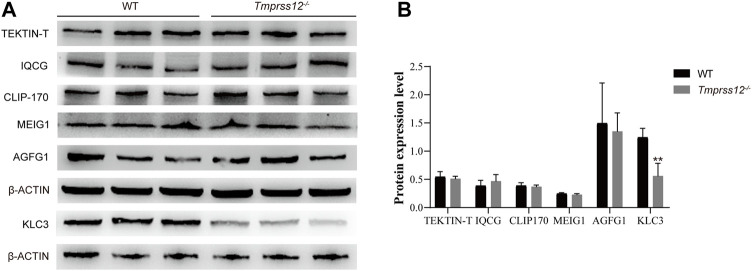
Mitochondrial development in testicular spermatids was impaired in *Tmprss12* knockout mice. **(A)** Western blot of special molecules involved in spermiogenesis from the testes of WT and *Tmprss12* ^−/−^ mice. β-ACTIN was used as an internal control. **(B)** Grey intensity analysis showing that the expression level of KLC3 decreased in *Tmprss12* ^−/−^ mice (*n* = 3). Data are the mean ± s.d. ^∗^ *p* < 0.05.

To investigate whether the abnormal mitochondrial structure is caused by damaged spermiogenesis, we detected special molecules involved in spermiogenesis in the testes, including TEKTIN-T, IQCG, CLIP-170, MEIG1, AGFG1, and KLC3. TEKTIN-T and IQCG are essential for sperm flagellum formation (Tanaka et al., 2004; Li et al., 2014). CLIP-170 and MEIG1 are critical for manchette structures (Akhmanova et al., 2005; Zhang et al., 2009). AGFG1 is involved in acrosomal development (Kang-Decker et al., 2001). KLC3 plays an important role in mitochondrial migration and sperm tail midpiece formation (Zhang et al., 2012). Real-time PCR and western blot both showed that the expression of KLC3 decreased in the testes of *Tmprss12* ^*−/−*^ mice (Figures 7A,B; Supplementary Figure 3).

### Absence of *Tmprss12* Affects the Expression of Some Proteins in Mouse Testes

**FIGURE 8:**
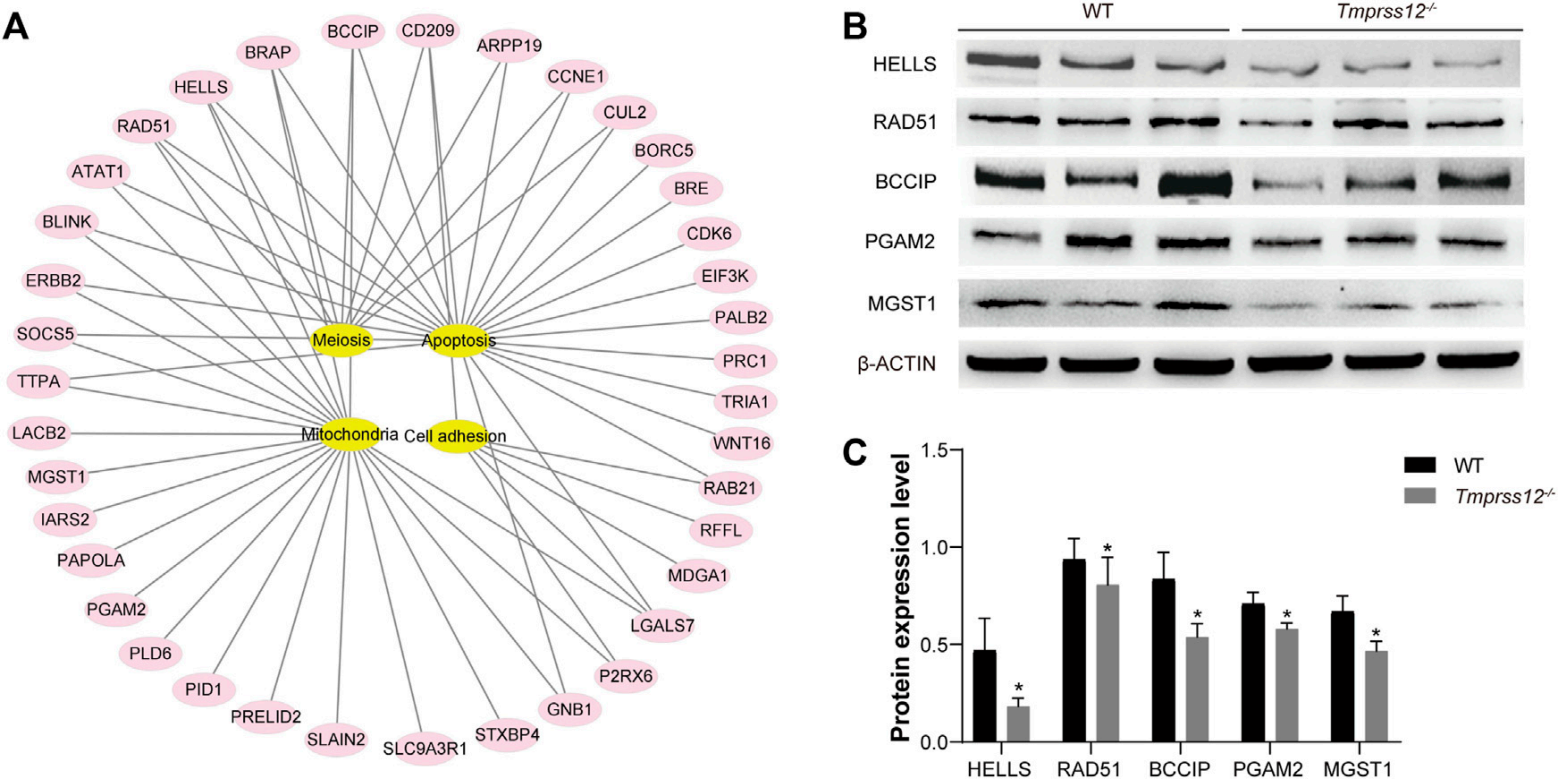
Analysis of differential protein expression profiles of testes from WT and *Tmprss12* ^−/−^ mice. **(A)** Proteins showing a difference of more than 2-fold were analysed according to IPA (ingenuity pathway analysis), and they were mainly related to meiosis, apoptosis, mitochondrial function and cell adhesion. **(B)** Verification of the expression levels of some differential proteins related to key events in WT and *Tmprss12* ^−/−^ mouse testes by western blot. β-ACTIN was used as an internal control. **(C)** Grey intensity analysis showing the expression level of the above proteins. The results were consistent with the differential expression profiles (*n* = 3). Data are the mean ± s.d. ^∗^ *p* < 0.05.

As a serine protease, TMPRSS12 should theoretically regulate target proteins to play a specific role in spermatogenesis. To further investigate the mechanism of TMPRSS12 in male reproduction, we used 2-D electrophoresis combined with MALDI-TOF-TOF MS/MS to construct differential protein expression profiles of testes in *Tmprss12* ^*−/−*^ and WT mice (Supplementary Figure 4). A total of 64 spots with 2-fold or more differential expression between two groups were identified using MALDI-TOF-TOF MS/MS. As a result, 54 unique proteins were identified successfully, including 39 proteins with decreased expression and 15 proteins with increased expression in *Tmprss12* ^*−/−*^ mice (Supplementary Table 2). We analysed the molecular functions of these differentially expressed proteins by IPA (Ingenuity Pathway Analysis) and found that these proteins were mainly related to meiosis, apoptosis, mitochondrial function and cell adhesion (Figure 8A).

To certify the expression levels of differential proteins, some proteins related to key events were selected for an expression analysis by western blot. For example, lymphoid-specific helicase (HELLS) has been proven to be essential for meiotic progression, especially in chromosomal synapses (Zeng et al., 2011; Kollarovic et al., 2020). Recombinase RAD51 is necessary for DSB repair and homologous recombination (Hinch et al., 2020). BCCIP interacts with RAD51 to stimulate DNA pairing activity in homologous recombination (Kelso et al., 2017). PGAM2 activity could modulate mitochondrial function (Okuda et al., 2013). MGST1 is a glutathione transferase located in mitochondrial membranes that protects mitochondria against oxidative stress to ensure their normal biological function (Zhang et al., 2001; Johansson et al., 2010; Schaffert, 2011). The western blot results showed that the expression trend of these proteins was consistent with the differential expression profiles (Figures 8B,C).

## Discussion

Mammalian reproduction is a complicated and finely regulated process. For males, spermatogenesis takes place in the testis, and the mature sperm must capacitate, migrate to the oviduct ampulla, penetrate the cumulus oophorus cells and zona pellucida and finally fuse with oocytes. Many testis-predominant proteins involved in the process have been studied to explore the biological characteristics and regulatory mechanism of spermatogenesis and sperm function.

Because it activates various proteins, serine protease is considered a relative upstream regulator in physiological processes (Bugge et al., 2009; Shen B. et al., 2013), and it has attracted the attention of researchers in various fields. Many serine protease members have been found to be involved in different stages of spermatogenesis and/or sperm function (Mbikay et al., 1997; Kawano, et al., 2010; Yoneda and Kimura, 2013; Yoneda et al., 2013; Shen C. et al., 2013; Shang et al., 2018; Kobayashi et al., 2020; Holcomb et al., 2020; Zhu et al., 2021). Compared with PRSS proteins, fewer TMPRSS members have been identified and studied. At present, only one member, TMPRSS12, has been found to be specifically expressed in the testis and sperm (Liu et al., 2013; Wang et al., 2013).

*Larasati* et al. first generated *Tmprss12* ^*−/−*^ mice and demonstrated that *Tmprss12* ^−/−^ males were sterile. They found that *Tmprss12* ^−/−^ mice had reduced sperm motility, ZP binding defects and low IVF rates and their spermatozoa failed to migrate through the uterotubal junction (Larasati et al., 2020). The study preliminarily confirmed that TMPRSS12 was related to sperm function. Similar to the above study, our results also confirmed that *Tmprss12* ^−/−^ mice were sterile and had low sperm motility and *in vitro* fertilization rates (Supplementary Figure 5).

In this study, we focused on the role of TMPRSS12 in testis spermatogenesis; therefore, we detailed the expression and location of TMPRSS12 in the testis. TMPRSS12 was first detected in the testes of 10-day-old mice, and its expression remained constant after 14 days of age. TMPRSS12 was expressed over a long time range in adult mouse testes from spermatocytes to spermatids. These results suggested that TMPRSS12 might function in meiosis and spermiogenesis in spermatogenesis. Using *Tmprss12* ^*−/−*^ mice, we studied spermatogenesis in the testis. First, the increased TUNEL-positive cells in spermatocytes and decreased counts of spermatogenic cells in *Tmprss12* ^−/−^ testes were observed, which could provide an explanation for the decreased sperm count observed in this study. The above data indicated that the loss of TMPRSS12 might influence the biological process of meiosis, which corresponds with its expression in spermatocytes. It is well known that meiosis is an important biological process in spermatogenesis. During meiosis, pairing of homologous chromosomes, synapsis, recombination and chromosome segregation are conserved events (Zickler and Kleckner, 1999; Page and Hawley, 2004). In WT pachynema, 19 pairs of autosomes and partial synapsed sex chromosomes in the pseudoautosomal region (PAR) were observed. However, we found that *Tmprss12* ^−/−^ mice had chromothripsis, no synapsed sex chromosomes, and abnormal SYCP1 signalling in non-PAR, thus demonstrating abnormal synapsis in these mice (de Vries et al., 2005). We also studied DSB repair during meiosis. DSBs are marked by the presence of phosphorylated H2AX (γH2AX) (Mahadevaiah et al., 2001). While γH2AX labelling successively disappeared from the autosomes and was restricted to the sex chromosomes in the pachynema of WT mice, the γH2AX signal remained and surrounded most of the chromosomes in a cloud-like manner in *Tmprss12* ^*−/−*^ mice, which indicated that DSBs were not efficiently repaired. Thus, our findings demonstrated that meiosis progress was arrested in some spermatocytes when TMPRSS12 was lost, which resulted in increased apoptosis of spermatocytes.

As a protease, TMPRSS12 theoretically plays its role by influencing the expression and function of some proteins. In this study, we explored the downstream proteins targeted by TMPRSS12 through proteomics methods to elucidate the mechanism. A series of potential targeted proteins involved in meiosis were identified, and some of them were verified by our experimental results, including HELLS, BCCIP, and RAD51. HELLS is a member of the SNF2 family of chromatin remodelling proteins. HELLS-deficient spermatocytes exhibit abnormal meiosis, including extensive asynapsis of the autosomal chromatin compartment and failure to form the sex body, resulting in pachytene spermatocyte arrest and apoptosis (Zeng et al., 2011; Kollarovic et al., 2020). The observed decrease in HELLS in *Tmprss12* ^*−/−*^ mice disturbed the synapsis of homologous chromosomes. Recombinase RAD51 and its paralog DMC1 localized at DNA DSB sites in the meiotic prophase and played a key role in the repair of DSBs (Hinch et al., 2020). BCCIP binds DNA and physically and functionally interacts with RAD51 to stimulate its homologous DNA pairing activity in homologous recombination (Kelso et al., 2017). We confirmed the decreased expression of BCCIP and RAD51 in *Tmprss12* ^−/−^ mice, which influenced the repair of chromosomal lesions, such as DSBs. Because homologous recombination is a DNA repair pathway, defects in this repair pathway may cause chromosomal aberrations and meiosis arrest.

Normal spermatogenesis in the testis not only ensures an adequate sperm count but also guarantees sperm quality. During the process of spermiogenesis, round spermatids undergo dramatic morphological, molecular and cellular alterations, thus laying a foundation for the generation of functional sperm (Ozturk et al., 2017; Zakrzewski et al., 2021). Due to its predominant expression in spermatids, TMPRSS12 very likely participates in spermiogenesis. Unique molecules involved in spermiogenesis were detected, and KLC3, which plays a role in the development of sperm tail midpieces and mitochondrial transport during spermiogenesis (Zhang et al., 2012), was found to have a low expression level in *Tmprss12* ^−/−^ mice. To explore the relationship between TMPRSS12 and mitochondria, we observed the morphology and ultrastructure of sperm. The *Tmprss12* ^−/−^ mice had a normal morphology, which is consistent with the findings of Larasati et al. (2020). Ultrastructure analysis showed the normal head shape and microtubule structure of the tail in *Tmprss12* ^−/−^ sperm, which were the same as those found by Larasati et al. However, we found blurred or absent inner mitochondrial membrane (IMM) cristaes in the sperm of *Tmprss12* ^−/−^ mice. By double-blind counting and statistical analysis, we demonstrated that the percentage of mitochondria with abnormal structures was significantly increased in *Tmprss12* ^−/−^ mice, and we speculated that mitochondrial function was disturbed. Thus, we measured the oxygen consumption level of sperm and detected several key markers related to ATP synthesis in the mitochondria, including COX2, COX3, MTATP and MTCYB (Feng et al., 2008; Heidari et al., 2016). The reduction in oxygen consumption and the decreased expression of MTATP and MTCYB suggested that the dysfunction of aerobic oxidation in mitochondria affected the energy production and motor function of sperm in *Tmprss12* ^−/−^ mice. In this study, we identified two potential targeted proteins (PGAM2 and MGST1) of TMPRSS12 that are related to the function of mitochondria (Zhang et al., 2001; Johansson et al., 2010; Schaffert, 2011; Okuda et al., 2013). However, the exact mechanism by which TMPRSS12 affects mitochondrial development and function needs to be explored further.

It is worth mentioning that MMP detection was also performed in our study, and a decrease in MMP was observed in *Tmprss12* ^*−/−*^ mice, indicating that the mitochondria were unable to maintain normal physiological functions to synthesize ATP. This result verified the influence of TMPRSS12 on mitochondrial function. In addition, we detected the apoptosis status of the sperm because a decline in MMP is an important event in early apoptosis. The results demonstrated that the expression levels of BCL-2 decreased and BAX and CASPASE3 increased, indicating that the deletion of TMPRSS12 might activate mitochondrial apoptotic pathways in sperm, which influence the counts of sperm in the epididymis. This is also one of the reasons for oligospermia in *Tmprss12* ^−/−^ mice.

Although this study focused on spermatogenesis, we also noticed the effect of TMPRSS12 on sperm function. Similar to the study by Larasati et al. (2020), our data showed that TMPRSS12 was expressed in the acrosomal region of sperm. This specific location in sperm suggested the potential role of TMPRSS12 in acrosomal reactions, sperm-egg interactions, etc. Larasati et al. interpreted the role of TMPRSS12 in uterotubal junction migration in mice. We performed additional research on sperm function. CTC staining results showed that the rate of sperm capacitation and the acrosome reaction of *Tmprss12* ^−/−^ mouse sperm decreased significantly (Supplementary Figure 6). In addition, *in vivo* and *in vitro* fertilization experiments were conducted. The results showed that the *in vivo* fertilization rate was 0 and the *in vitro* fertilization rate was severely reduced in *Tmprss12* ^−/−^ mice (Supplementary Figures 5, 7), suggesting that the decreased sperm motility and the impaired sperm-egg interaction synergically affected the fertilization results of these mice. It is worth noting that Larasati et al. found very little expression of mature ADAM3 in *Tmprss12* KO spermatozoa (Larasati et al., 2020). Since ADAM3 is an essential protein for sperm-egg interactions (Inoue et al., 2011), this result also supports the involvement of TMPRSS12 in sperm-egg interactions. However, the mechanism of TMPRSS12 in regulating sperm function requires further investigation.

In summary, we confirmed the important role of TMPRSS12 in spermatogenesis and preliminarily identified its potential targets. By affecting specific molecules, TMPRSS12 regulates chromosomal synapse and DSB repair during spermatocyte meiosis and influences mitochondrial development during spermiogenesis, resulting in decreased sperm counts and quality. Our study provides a more complete explanation for male sterility in *Tmprss12* ^−/−^ mice. The involvement of TMPRSS12 in many aspects of male reproduction suggests its key role. Considering the oligospermia, asthenospermia and infertility phenotype exhibited by *Tmprss12* ^−/−^ mice and the deleterious missense mutation of *Tmprss12* found in infertile men with dyszoospermia, *Tmprss12* is likely to be a potential pathogenic gene in these patients. TMPRSS12 and its downstream molecules could become potential targets for the clinical diagnosis and treatment of infertility. In the future, further research is required to confirm whether potential deleterious mutations affect TMPRSS12 protein function.

## Data Availability

The datasets presented in this study can be found in online repositories. The data presented in the study are deposited in the PRIDE repository, accession number PXD028014.
