# Supplementary material for: TMPRSS12 Functions in Meiosis and Spermiogenesis and Is Required for Male Fertility in Mice
Source: Front Cell Dev Biol. 2022 Apr 25;10:757042. doi: 10.3389/fcell.2022.757042 (PMC9081376; doi:10.3389/fcell.2022.757042)
Supplement: Supplementary file 1 [file DataSheet1.PDF]

Supplementary figures

gene	transcript	codon_change	aa_change	aa_length	impact	prediction	aaf_1kg_eas	aaf_1kg_all	aaf_adj_exac_all	aaf_adj_exac_eas	num_het	num_hom_alt
TMPRSS12	ENST00000398458	c. 65A>G	p. His22Arg	348	missense_variant	harmless	-1	-1	-1	-1	1	0
TMPRSS12	ENST00000398458	c. 157C>T	p. Arg53Trp	348	missense_variant	harmless	-1	-1	-1	-1	1	0
TMPRSS12	ENST00000398458	c. 262G>T	p. Ala88Ser	348	missense_variant	harmless	-1	-1	0. 000163497	0. 002938296	1	0
TMPRSS12	ENST00000398458	c. 466G>A	p. Ala156Thr	348	missense_variant	harmless	0	0. 000199681	6. 21E-05	0	1	0
TMPRSS12	ENST00000398458	c. 634G>C	p. Gly212Arg	348	missense_variant	deleterious	-1	-1	-1	-1	1	0
TMPRSS12	ENST00000398458	c. 994C>T	p. Arg332Cys	348	missense_variant		0	0. 000399361	5. 80E-05	0	1	0

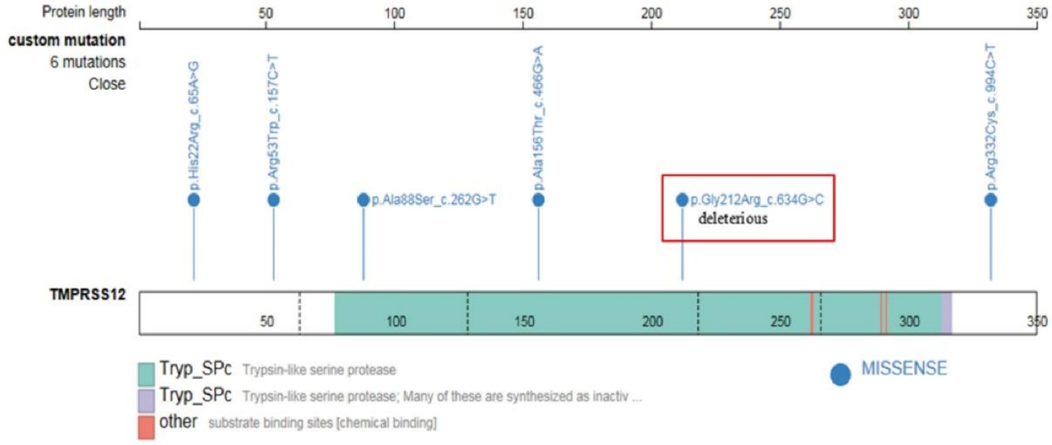

Figure S1. Identification of the potential deleterious missense mutations of *Tmprss12* gene in dyszoospermia patients.

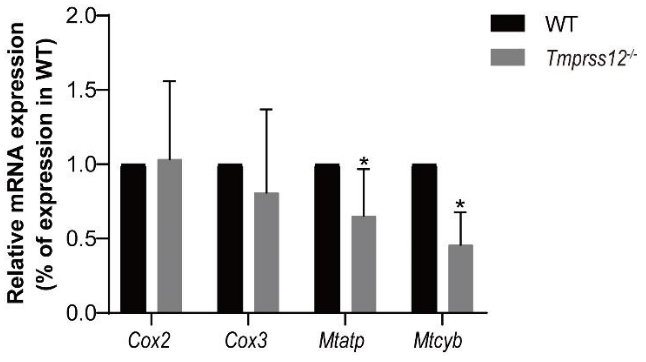

Figure S2. Detection of the mRNA expression levels of key markers in the mitochondrial electron transfer chain in sperm from WT and *Tmprss12*$^{-/-}$ mice. (n=3). Data are the mean±s.d. $^{*}P < 0.05$.

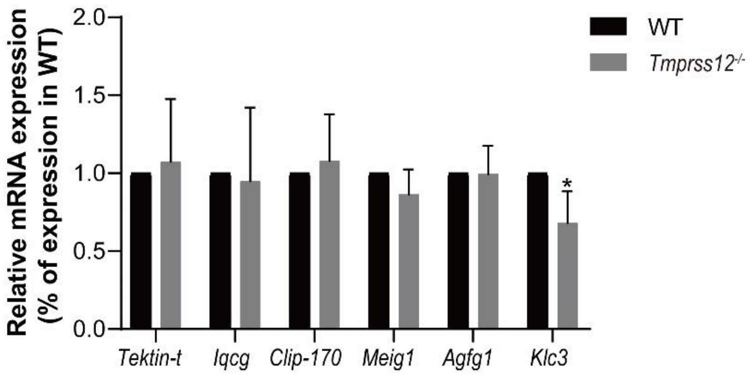

Figure S3. Detection of the mRNA expression levels of special molecules involved in spermiogenesis from testes of WT and *Tmprss12*$^{-/-}$ mice. (*n*=3). Data are the mean±s.d. $^{*}P < 0.05$.

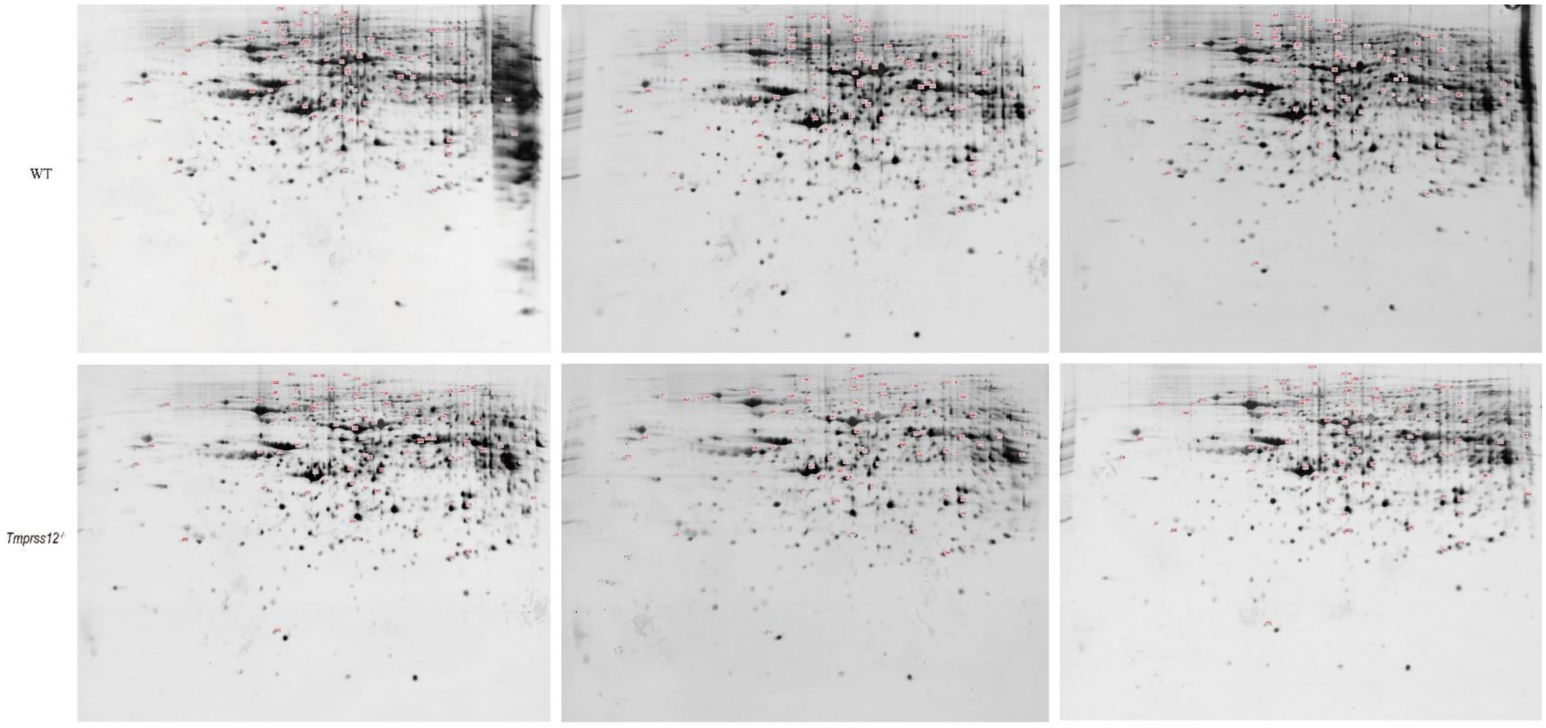

Figure S4. Silver-stained reference 2-DE map of testes proteins from WT and *Tmprss12*$^{-/-}$mice. (*n*=3).

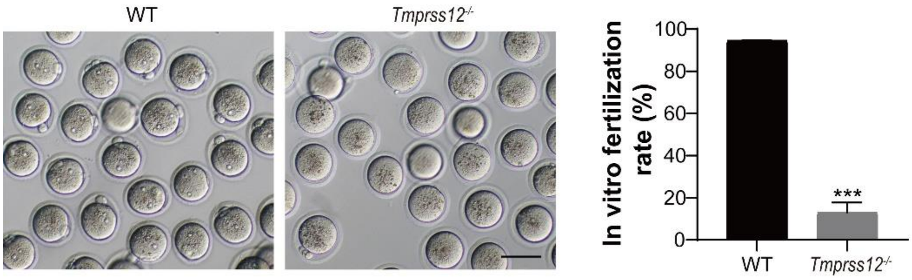

Figure S5. Analysis of the in vitro fertilization ability of sperm from WT and *Tmprss12*$^{-/-}$ mice. (*n*=3). Scale bar: 20 µm. Data are the mean±s.d. $^{***}P < 0.001$.

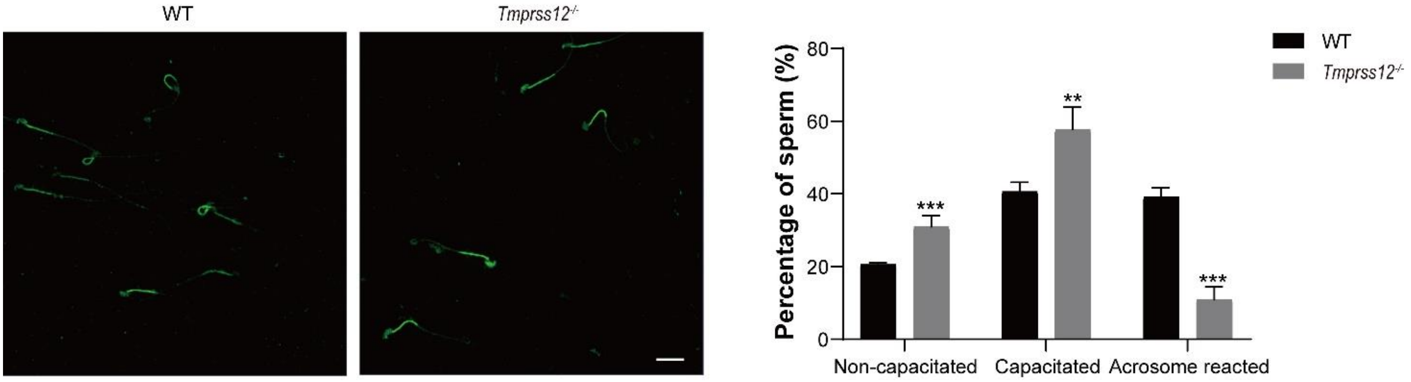

Figure S6. Assessment of capacitation and the acrosome reaction. (*n*=3). Scale bar: 20 µm. Data are the mean±s.d. $^{**}P < 0.01$, $^{***}P < 0.001$.

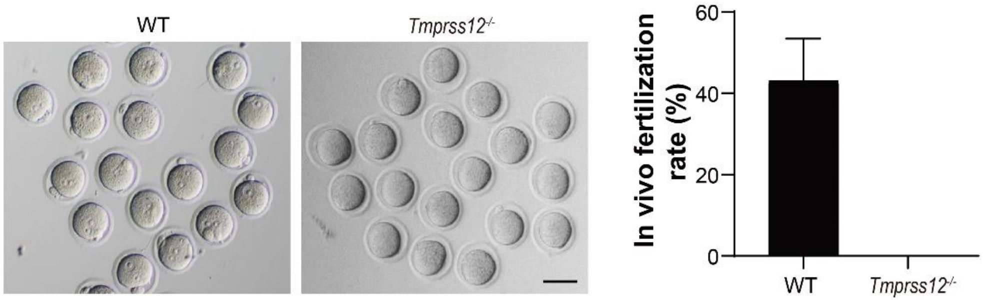

Figure S7. Analysis of the in vivo fertilization ability of sperm from WT and *Tmprss12*$^{-/-}$ mice. (*n*=3). Scale bar: 20 µm. Data are the mean±s.d.

Table S1. Primer sequences and target fragment size of each gene

Gene Symbol	Forward Primer	Reverse Primer	PCR Product (bp)
Tmprss12	CGGTCACGAAGGATTGTGGA	CTCCGGGTGAGGTCATTGG	259
Tektin-t	AACCAGGCTCCAGGGCTTTGT	ACTTGTCCAGCGTCTCCTTCCA	367
Iqcg	GCAAGGAGAAGGAGAAGAG	TGGAGCGAGAAGAAGGAT	243
Clip-170	AGATAGCGGCAGCAGACAAGGA	AGCAGTGGACAGAGGTGAAGCA	500
Meig1	GGCCAGAGACAGGGTACGTGAA	GGAGCATTGCCAAGCCGAAGAA	150
Agfg1	ACCTGAAGATGCTGCGGGACAT	GCTTGCTCTGGCGGAACATACC	361
Klc3	CGTGCTCTGGAGATCCGTGAGA	CTGCTGTGTATCGCCTGCTGTG	327
Cox2	CCCTTCCTCCCGTAGCAGATGA	ATCAGACCAGGCACCAGACCAA	445
Cox3	CACCAGTCAATCCCTGTTGTTACT	GGTAGTTGTCGAGGCCAAAGC	75
Mtatp6	GCAGAGCATGACGGACGAGTTC	CCACCCACCCACCTACACCAAA	169
Mtcyb	CTCACTTGCCCACTTCCTTC	GTAAGCCGGACTGCTAATGC	114
Actin	AGATCAAGATCATTGCTCCTCCT	ACGCAGCTCAGTAACAGTCC	174

Table S2. Fifty-four proteins showing an expression difference of 2-fold or more

Number	Group ID	Protein	FoldChange (WT/Homo)
1	83	BCCIP_MOUSE	6489.43
2	103	LACB2_MOUSE	3614.225
3	121	AWAT2_MOUSE	916.61
4	130	WNT16_MOUSE	848.971
5	71	RAD51_MOUSE	727.497
6	60	RFFL_MOUSE	496.758
7	85	BRE_MOUSE	481.902
8	52	ATAT_MOUSE	377.918
9	70	TTPA_MOUSE	356.5509
10	106	GBB1_MOUSE	346.0064
11	154	HELLS_MOUSE	331.7065
12	89	BLNK_MOUSE	295.8272
13	127	STXB4_MOUSE	268.6801
14	140	BRAP_MOUSE	184.023
15	147	MATN4_MOUSE	170.9588
16	78	NHRF1_MOUSE	128.2715
17	1244	MDGA1_MOUSE	11.61092681
18	153	PRC1_MOUSE	9.684779365
19	1243	SYIM_MOUSE	9.616219949
20	97	PRS54_MOUSE	5.169462863
21	393	PRLD2_MOUSE	4.849559841
22	159	PALB2_MOUSE	4.597226529
23	1004	TACD2_MOUSE	4.496940192
24	355	IN80C_MOUSE	4.480933845
25	716	ATGA1_MOUSE	4.464323264
26	786	DCD2C_MOUSE	4.358446106
27	1174	NR1D2_MOUSE	4.200867981
28	1222	PGAM2_MOUSE	3.922478226
29	1247	PAPOA_MOUSE	3.902966755
30	1173	ADA15_MOUSE	3.466688421
31	66	MGST1_MOUSE	3.193873865
32	780	PCLI1_MOUSE	3.130903979
33	1171	SOCS5_MOUSE	3.027887372
34	1003	P2RX6_MOUSE	2.913851
35	1139	SLAI2_MOUSE	2.753277762
36	160	TRIA1_MOUSE	2.723319532
37	1203	ERBB2_MOUSE	2.273884008

38	437	FCOR_MOUSE	2.2273608
39	924	T3JAM_MOUSE	2.15767242
40	578	PLD6_MOUSE	0.45395122
41	627	BORC5_MOUSE	0.37810162
42	1024	ASB8_MOUSE	0.35531349
43	1021	GCSAM_MOUSE	0.342763655
44	732	RAB21_MOUSE	0.322968886
45	917	CDK6_MOUSE	0.30546563
46	960	MP2K5_MOUSE	0.245154765
47	956	EIF3K_MOUSE	0.232514073
48	100	CCNE1_MOUSE	0.220298693
49	671	PR2C2_MOUSE	0.213766058
50	725	C209E_MOUSE	0.153878407
51	975	LEG7_MOUSE	0.091861476
52	1109	CUL2_MOUSE	0.00504079
53	1123	JKIP1_MOUSE	0.003725533
54	344	ARP19_MOUSE	0.000575527
